# Lack of Relationship between Cord Serum Angiopoietin-Like Protein 4 (ANGPTL4) and Lipolytic Activity in Human Neonates Born by Spontaneous Delivery

**DOI:** 10.1371/journal.pone.0081201

**Published:** 2013-12-04

**Authors:** Henar Ortega-Senovilla, Ute Schaefer-Graf, Katrin Meitzner, Kristof Graf, Michael Abou-Dakn, Emilio Herrera

**Affiliations:** 1 Department of Chemistry and Biochemistry, Faculties of Pharmacy and Medicine, Universidad San Pablo-CEU, Madrid, Spain; 2 Department of Obstetrics and Gynecology, Berlin Center for Diabetes in Pregnancy, St. Joseph's Hospital, Berlin, Germany; 3 Department of Cardiology, Jewish Hospital, Berlin, Germany; University of Basque Country, Spain

## Abstract

**Background:**

Ligands of peroxisome-proliferator activated receptors (PPARs), such as non-esterified fatty acids (NEFAs), induce expression of angiopoietin-like protein 4 (ANGPTL4). Recently ANGPTL4 has been reported to be a mediator of intracellular adipose lipolysis induced by glucocorticoids.

**Objective:**

To determine the concentrations of ANGPTL4 in cord serum of neonates born by spontaneous vaginal delivery (SVD) and by pre-labor cesarean section (CS) from healthy women, and to relate them to parameters of neonatal lipolytic activity at birth.

**Measurements:**

In 54 neonates born by SVD and in 56 neonates born by CS, arterial cord blood was drawn to determine insulin, cortisol, triacylglycerols (TAGs), glycerol, non-esterified fatty acids (NEFAs), individual fatty acids, ANGPTL4, adiponectin, retinol binding protein 4 (RBP4) and leptin.

**Results:**

Birth weight and neonatal fat mass in SVD and CS showed no difference, but the concentrations of glycerol, adiponectin, RBP4, NEFAs and most individual fatty acids were higher in cord serum of neonates born by SVD compared to CS, indicating a higher adipose tissue breakdown in the SVD group. The concentrations of TAG and cortisol were also higher and that of insulin was lower in cord serum of SVD compared to the CS group. However, the concentration in cord serum of ANGPTL4 did not differ between the two groups and no positive correlation with either NEFA or glycerol concentrations were detected.

**Conclusion:**

ANGPTL4 is known to stimulate lipolysis in adults, but does not appear to mediate the increased activity in SVD, indicating the presence of different regulatory inputs.

## Introduction

Basal and hormone-stimulated triacylglycerol (TAG) lipolysis and efflux of fatty acids from adipose tissue is a complex process involving multiple regulatory factors [1], [2], structural proteins [3] and carrier proteins [4], but the way in which adipocytes integrate all these signals is not completely understood. The intracellular hydrolysis of TAG is facilitated by multiple hydrolases localized near the surface of the lipid droplet [5]; the activity is controlled by a coordinated mechanism, that involves the phosphorylation of the hydrolases by cAMP-dependent protein kinase A in response to various signals [6]. Recently it has been reported that angiopoietin-like protein 4 (ANGPTL4) is a physiological mediator of intracellular adipose tissue lipolysis induced by catecholamines and glucocorticoids: it acts upstream of adenylate cyclase and downstream of receptor activation and stimulates the production of cAMP [7], [8].

ANGPTL4 is synthesized primarily in liver and adipose tissue [9]–[11]. The main role attributed to ANGPTL4 has been its action as an inhibitor of lipoprotein lipase [12]–[14]; indeed, in mice, over-expression of ANGPTL4 does cause hypertriacylglycerolemia [15] and inactivation of its gene results in hypotriacylglycerolemia [12], [13]. However, in adipose tissue of fasted ANGPTL4^−/−^ mice, the concentrations of cAMP and the absolute abundance of phosphorylated forms of hormone sensitive lipase (HSL) and perilipin were lower than in wild type controls [7], indicating that ANGPTL4 is needed for cAMP-dependent protein phosphorylation and the activation of lipolysis. In addition, a positive correlation between ANGPTL4 expression levels and the mRNA expression levels of HSL have been found in human adipose tissue [16]. Thus, in view of the reported relationships with the stimulation of fat mobilization, the role of ANGPTL4 in the regulation of blood lipids levels may be greater than initially ascribed.

ANGPTL4 gene expression is regulated by several signals, including fasting and glucocorticoids [8]. Interestingly, it has been shown that non-esterified fatty acids (NEFA) induce ANGPTL4 gene expression via their interaction with peroxisome proliferator-activated nuclear receptors (PPAR) [17], [18]. Consistent with this is the recent report that glucocorticoids increase and insulin decreases circulating ANGPTL4 concentrations in humans, effects which are secondary to the actions of these hormones on NEFA concentrations [19]. Thus through its stimulation of adipose tissue lipolysis, the increase in ANGPTL4 concentration could raise serum NEFA, which could stimulate the expression of more ANGPTL4 as part of a positive feedback loop, especially important in situations of prolonged high energy demand.

Physiologically, spontaneous delivery is a condition where there is a decline in circulating insulin and an increase in serum catecholamines and cortisol concentrations in the newborn [20]–[24]. The effects of these hormones on adipose tissue lipolytic activity are well-known [25], [26], so spontaneous delivery increases the mobilization of fetal lipid reserves, increasing the concentrations of circulating lipolytic products, NEFA and glycerol. Maternal analgesia during labor does not alter these variables in fetal circulation, supporting a stress response in the fetus that is independent of that in the mother [22], [27]. In contrast, delivery by pre-labor caesarean section takes place without a similar neonatal lipolytic stimulus [20]. Thus, by comparing concentrations of ANGPTL4 in serum and other parameters related to lipolytic activity in neonates born by spontaneous delivery and comparing them to those born by pre-labor caesarean section, it was possible to test the hypothesis that ANGPTL4 plays a role in regulating neonatal lipolytic activity. Results show that the different lipolytic activities occurring in these two conditions are not associated with changes in ANGPTL4 concentrations, indicating that this protein does not contribute to the higher lipolytic activity normally taking place in spontaneous delivery.

## Research and Design Methods

### Study subjects

The neonates from 110 uncomplicated pregnancies were studied. Fifty four were born by spontaneous vaginal delivery (SVD) and fifty six by scheduled pre-labor cesarean section (CS). Indications for elective CS were previous cesareans, suspected macrosomia, suspected feto-pelvic disproportion, breech presentation, suspected intrauterine growth retardation with or without suspected fetal Doppler indices, and maternal request. Both populations were recruited from women who gave birth at the Department of Obstetrics of the Vivantes Medical Center in Berlin. All the women showed normal 75 g oral glucose tolerance test (OGTT) according to Carpenter & Coustan criteria (90/180/155 mg/dl) [28] at 27.1±3.5 weeks of gestation, and those with maternal diseases known to affect fetal growth, such as gestational diabetes, maternal alcohol or drug consumption, autoimmune and endocrine diseases, preeclampsia, chronic hypertension or pregnancy-induced hypertension were excluded. Emergency caesarean cases were also excluded from the study. Gestational age was calculated from the last menstrual period and confirmed by an ultrasound examination performed before 20 weeks of gestation. All pregnancies were singleton and none of the babies exhibited malformations, abnormal karyotypes or signs of distress at delivery. Birth weight and body length were obtained shortly after delivery, and neonatal skinfold thickness at the flank was measured within 48 hours. Neonatal fat mass was calculated by a formula derived from Catalano et al. [29]. Infants with birth weight <10th percentile were classified as small for gestational age (SGA), and those with birth weight >90th percentile as large for gestational age (LGA) based on gestational age and sex adjusted birth weight percentiles derived from German national database [30]. The study was approved by the Vivantes Medical Center of Berlin ethics committee and all the women gave written informed consent.

### Sampling

Cord blood samples from one of the umbilical arteries were obtained from a segment of the cord immediately after delivery. Blood samples were centrifuged (1500×g at 4°C for 25 min) and serum was divided into small aliquots and immediately stored at −80°C until analysis. None of the samples used in the study were hemolysed.

### Analytical methods

Serum cholesterol, TAG (Menarini Diagnostics, Florence, Italy), glycerol and NEFA (Wako Chemical, Neuss, Germany) were determined enzymatically using commercial kits. HDL-cholesterol (HDL-c) was measured after precipitation of apoB-containing lipoproteins with phosphotungstic acid and magnesium (Boehringer-Mannheim, Ingelheim, Germany). LDL-cholesterol (LDL-c) was calculated according Friedewald's formula [31].

Serum cortisol, ANGPTL4, insulin, leptin, adiponectin and retinol-binding protein 4 (RBP4) concentrations were measured using ELISA kits according to the manufacturer's instructions. Specifically, cortisol was measured using a competitive ELISA kit (DRG International, New Jersey, USA; intra-assay variation 3.2% and inter-assay variation 7.7%), ANGPTL4 were determined using sandwich ELISA kit (USCN Life Science, Wuhan, China), with intra-assay variations of <10% and inter-assay variation of <12%, insulin, adiponectin and leptin were measured using sandwich ELISA kits (Mercodia AB, Uppsala, Sweden; intra-assay variation 3.4%, 3.0% and 2.3% and inter-assay variation 3.6%, 5.3% and 5.2%, respectively) and RBP4 was measured using a sandwich ELISA kit (AdipoGen, Seoul, Korea; intra assay variation 3.4% and inter assay variation 7.1%).

For the analysis of fatty acid profiles, serum lipids were extracted in chloroform/methanol (2∶1) [32] containing 0.005% BHT and an internal standard of nonodecenoic acid (19∶1). Dried lipid extracts were subjected to methanolysis for 2.5 h at 80°C in methanol:toluene (4∶1) containing acetyl chloride and the methyl esters were analyzed on a Perkin Elmer gas chromatograph (Autosystem; Norwalk, CT) in the presence of methyl-heptadecanoate (17∶0) as a reference standard, as previously reported [33].

### Statistics

Results are expressed as means ± SEM. Statistical differences between groups were determined by analysis of variance (ANOVA) after adjusting for maternal pre-pregnancy body mass index (BMI), gestational age, sex and neonatal fat mass. When differences were statistically significant, multiple comparisons were performed using Tukey's post hoc test. Given their skewed distributions, concentrations of cortisol, insulin, TAG, NEFA, glycerol, adiponectin, RBP4, leptin, ANGPTL4 and polyunsaturated fatty acids (PUFAs) were log-transformed before statistical comparison. Correlations were tested by Pearson's method using the log-transformed data as indicated. To ascertain the independent predictors of NEFA, stepwise multiple regressions with backward selection were performed. All statistical analysis was performed using a computer software package (Statgraphics Centurion XV, version 15.2.06, Statistical Graphics Corp., Princeton, New Jersey).

## Results

As shown in Table 1, women having spontaneous deliveries were younger than those undergoing caesarean sections, but neither maternal pre-pregnancy BMI nor BMI at delivery differed between the studied groups. No significant differences in the neonatal-birth weight, fat mass or neonatal fat mass as a percentage of birth weight were observed between the two groups, or in the proportion of SGA and LGA neonates, placental weight, neonatal sex distribution, or Apgar score at 5 and 10 minutes. Only 2 neonates in SVD and 3 in the CS group showed an Apgar score at 5 minutes lower than 7, and the Apgar score at 10 minutes was lower than 7 in just one neonate of the CS group. Values of serum pH were all within the normal range, indicating no fetal distress, although neonates born by SVD showed slightly lower values.

**Table 1:** Characteristics of mothers and their offspring.

	Spontaneous delivery	Caesarean section	
	(n = 54)	(n = 56)	*P*
MATERNAL CHARACTERISTICS			
Age (years)	29.0±0.7	31.3±0.7	0.0209
Pre-pregnancy BMI (kg/m^2^)	25.1±0.6	25.5±0.6	n.s.
Glucose fasting OGTT (mg/dL)	75.0±1.2	77.4±1.2	n.s.
Glucose 1 h post OGTT (mg/dL)	135±3	131±3	n.s.
Glucose 2 h post OGTT (mg/dL)	103±3	105±3	n.s.
Gestational age (weeks)	38.8±0.1	38.5±0.1	n.s.
Delivery BMI (kg/m^2^)	30.2±0.6	30.7±0.6	n.s.
NEONATAL CHARACTERISTICS			
Birth weight (kg)	3.42±0.06	3.28±0.06	n.s.
Fat mass (g)	401±22	364±22	n.s.
% (FM/BW)	11.1±0.5	10.5±0.5	n.s.
SGA % (n)	13 (7)	9 (5)	n.s.
LGA % (n)	16 (8)	12 (7)	n.s.
Placenta weight (g)	628±16	659±16	n.s.
Female sex % (n)	50.9 (27)	49.1 (27)	n.s.
APGAR 5	9.65±0.10	9.40±0.10	n.s.
APGAR 10	9.85±0.06	9.79±0.06	n.s.
pH arterial	7.24±0.01	7.27±0.01	0.0009

All values are mean±S.E.M. OGTT, oral glucose tolerance test; FM, neonatal fat mass; BW, neonatal birth weight; SGA, small for gestational age; LGA, large for gestational age.

The concentrations of several metabolic parameters in cord serum after adjusting for pre-pregnancy BMI, gestational weeks at birth, sex and neonatal fat mass, are shown in Table 2. Cord serum cortisol and insulin varied according to the delivery mode, values for cortisol being higher in the spontaneous delivery neonates than in those of the CS group, while the concentrations of insulin showed the opposite trend. In accordance with the concentrations of cortisol and insulin, the concentrations of TAG, NEFA, glycerol and NEFA/glycerol ratio were also higher in umbilical arterial serum of neonates born by SVD than in those delivered by CS. In addition the concentrations of adiponectin and RBP4 were higher in SVD than in CS groups, but not so the concentrations of leptin and ANGPTL4, which were similar in the two groups. Also, neither the concentration of total cholesterol nor HDL-c and LDL-c differed between the two groups.

**Table 2:** Metabolic parameters in cord arterial serum of neonates born by spontaneous delivery and cesarean section.

	Spontaneous delivery	Caesarean section	
	(n = 54)	(n = 56)	*P*
Cortisol (ng/mL) ^(1)^	276±13	170±13	0.0000
Insulin (µU/mL) ^(1)^	4.51±0.66	8.34±0.66	0.0000
Triacylglycerol (mM) ^(1)^	0.407±0.015	0.327±0.015	0.0000
NEFA (µM) ^(1)^	103±6	48.7±6.1	0.0000
Glycerol (µM) ^(1)^	96.9±6.1	69.7±6.1	0.0043
NEFA/Glycerol (mol/mol) ^(1)^	1.23±0.10	0.87±0.10	0.0001
Total cholesterol (mg/dL)	64.7±2.5	59.1±2.5	n.s.
HDL-c (mg/dL)	26.5±1.2	24.4±1.2	n.s.
LDL-c (mg/dL)	21.6±1.0	19.0±1.0	n.s.
Adiponectin (µg/mL) ^(1)^	21.2±1.1	18.4±1.1	0.0372
RBP4 (µg/mL) ^(1)^	60.1±3.6	50.8±3.6	0.0381
Leptin (ng/mL) ^(1)^	11.3±1.7	12.2±1.9	n.s.
ANGPTL4 (ng/mL) ^(1)^	28.2±2.4	25.8±2.3	n.s.

All values are mean±S.E.M. adjusted for maternal BMI pre-pregnancy, gestational age, sex and neonatal fat mass. ^(1)^ Log-transformed skewed data were used for statistical comparisons. NEFA: non-esterified fatty acids; RBP: retinol binding protein; ANGPTL4: angiopoietin-like protein 4.

When the concentrations of individual fatty acids were determined in umbilical arterial serum (Table 3), it was found that their concentrations (adjusted for confounding variables as above) were always higher in neonates of SVD than in those that had been born by CS, with the single exception of eicosapentaenoic acid (20∶5 n-3), which had similar and low concentrations in both groups. The difference between the two groups was also significant when total saturated-, monounsaturated-, n-3- or n-6- polyunsaturated- fatty acids were determined, values always being higher in neonates of SVD than in those that had been delivered by CS. Moreover, most of these fatty acids showed a significant correlation with the concentration of glycerol and NEFAs in neonates of SVD but not in pre-labor CS (Table 4).

**Table 3:** Concentrations of fatty acids (mg/L) in cord arterial serum of neonates born by spontaneous delivery and cesarean section.

Fatty acid (mg/L)	Spontaneous delivery	Caesarean section	
	(n = 54)	(n = 56)	*P*
Palmitic acid (16∶0)	357±11	300±11	0.0003
Stearic acid (18∶0)	130±4	111±4	0.0012
Total SFA	530±17	445±16	0.0004
Palmitoleic acid (16∶1 n-7)	60.3±2.5	47.4±2.4	0.0003
Oleic acid (18∶1 n-9)	271±9	223±9	0.0002
Total MUFA	336±11	273±11	0.0001
α-linolenic acid (18∶3 n-3) ^(1)^	1.67±0.17	1.08±0.17	0.0330
Eicosapentaenoic acid (20∶5 n-3) ^(1)^	5.51±0.52	4.32±0.51	n.s.
Docosahexaenoic acid (22∶6 n-3) ^(1)^	72.3±3.1	61.4±3.0	0.0161
Total n-3 FA ^(1)^	79.5±3.4	66.8±3.3	0.0123
Linoleic acid (18∶2 n-6) ^(1)^	141±5	114±5	0.0002
γ-linolenic acid (18∶3 n-6) ^(1)^	4.53±0.22	3.76±0.21	0.0147
Dihomo γ-linolenic acid (20∶3 n-6) ^(1)^	42.0±1.3	35.7±1.3	0.0009
Arachidonic acid (20∶4 n-6) ^(1)^	172±7	149±6	0.0205
Total n-6 FA ^(1)^	381±12	321±12	0.0011
Total PUFA ^(1)^	461±15	388±15	0.0013

All values are mean±S.E.M. Adjusted for maternal BMI pre-pregnancy, gestational age, gender and neonatal fat mass. ^(1)^ Log-transformed skewed data were used for statistical comparisons. FA: fatty acids; SFA: saturated fatty acids; MUFA: monounsaturated fatty acids; PUFA, polyunsaturated fatty acids.

**Table 4:** Correlations coefficients of glycerol (µM) and NEFAs (µM) with concentrations of fatty acids (mg/L) in cord arterial serum of neonates born by spontaneous delivery and cesarean section.

	Spontaneous delivery		Cesarean section	
	(n = 54)		(n = 56)	
	Glycerol(1)	NEFA(1)	Glycerol(1)	NEFA(1)
Palmitic acid (16∶0)	0.0001	0.0122	0.1914	0.0566
Stearic acid (18∶0)	0.0002	0.0101	0.0453	0.0476
Total SFA	0.0001	0.0093	0.1362	0.0558
Palmitoleic acid (16∶1 n-7)	0.0164	0.4316	0.8382	0.2782
Oleic acid (18∶1 n-9)	0.0146	0.1363	0.5408	0.0569
Total MUFA	0.0121	0.1627	0.5860	0.0768
α-linolenic acid (18∶3 n-3) (1)	0.6281	0.5623	0.6285	0.2309
Eicosapentaenoic acid (20∶5 n-3) (1)	0.1094	0.1035	0.3938	0.5863
Docosahexaenoic acid (22∶6 n-3) (1)	0.0082	0.0559	0.2580	0.2526
Total n-3 FA (1)	0.0101	0.0507	0.2791	0.2541
Linoleic acid (18∶2 n-6) (1)	0.0028	0.0077	0.7219	0.1105
γ-linolenic acid (18∶3 n-6) (1)	0.1812	0.0330	0.4894	0.1522
Dihomo γ-linolenic acid (20∶3 n-6) (1)	0.1984	0.5291	0.9215	0.2732
Arachidonic acid (20∶4 n-6) (1)	0.0022	0.0054	0.3728	0.0751
Total n-6 FA (1)	0.0027	0.0093	0.5270	0.0724
Total PUFA (1)	0.0029	0.0115	0.4347	0.0834

(1) Log-transformed skewed data were used for statistical comparisons. FA: fatty acids; SFA: saturated fatty acids; MUFA: monounsaturated fatty acids; PUFA, polyunsaturated fatty acids.

**Figure 1:**
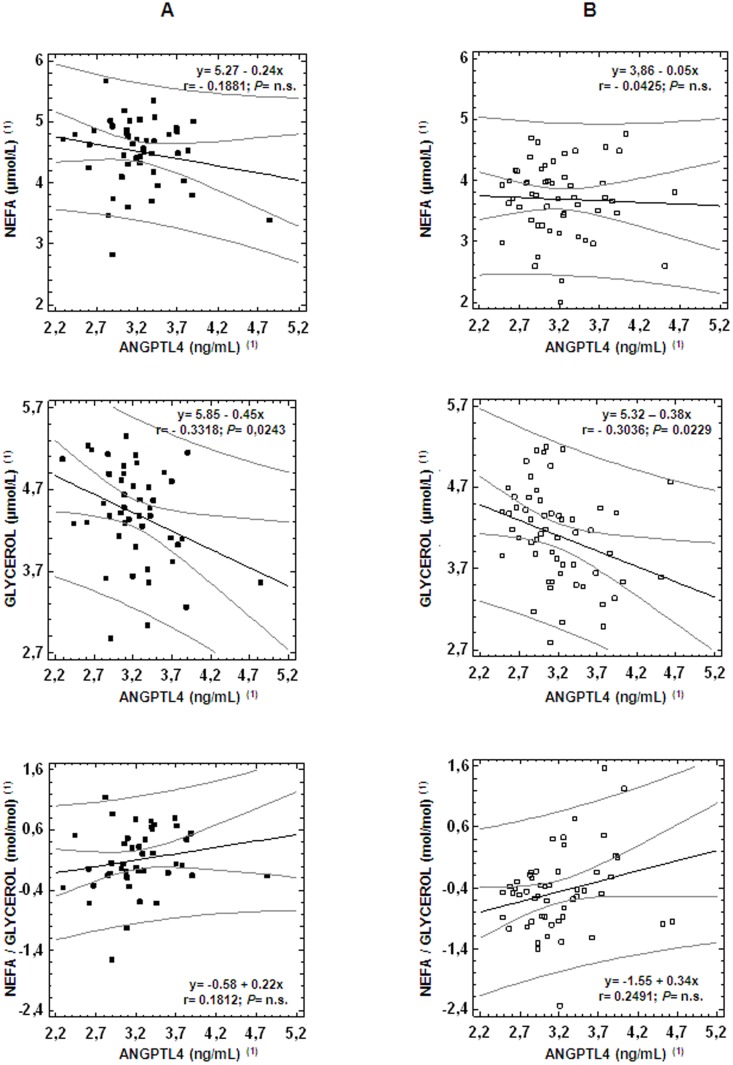
Correlations of ANGPTL4 with NEFA, glycerol and NEFA/glycerol ratio in cord serum. A. Neonates born by spontaneous delivery. B. Neonates born by cesarean section. In both, the regression line, confidence limits lines at 95%, prediction limits lines and Pearson correlation coefficients are shown. ^(1)^ Log-transformed skewed data were used for statistical comparisons

In order to test the hypothesis that a relationship between serum ANGPTL4 and NEFA or glycerol concentrations and its ratio exists, simple regression analysis was performed in spontaneous delivery and cesarean section groups separately. As shown in Figure 1, significant inverse linear correlations were found between cord serum ANGPTL4 and glycerol in both groups, whereas there were no significant correlations between ANGPTL4 and NEFA concentrations or the NEFA/glycerol ratio. Multiple regression analysis confirmed these results (data not shown).

## Discussion

The study reported herein showed that the concentration of ANGPTL4 in umbilical artery serum of neonates born by spontaneous delivery was similar to those found in neonates delivered by pre-labor cesarean section, in spite of the fact that the concentrations of NEFA, glycerol, and individual fatty acids as well as the NEFA/glycerol ratio were higher in the spontaneous delivery group. This finding indicated that, at least in gestations resulting in spontaneous vaginal delivery, there was no association of neonatal ANGPTL4 with the higher lipolytic products in adipose tissue that accompanies the onset of labor, delivery and the initial stages of neonatal adaptation.

It has been proposed that ANGPTL4 could act as a signal to prevent fat storage and stimulate intracellular adipose tissue lipolysis, especially in the situations of high energy demand, when ANGPTL4 could develop a key role in increasing the availability of fatty acids for β-oxidation [15]. These circumstances occur at birth, which marks the time when the newborn infant begins to use its own energy stores in order to regulate homeothermy, reduce peripheral utilization of glucose and supply energy to maintain vital neonatal functions. For spontaneous deliveries, fetal adipose tissue switches at a late stage of pregnancy to a higher lipolytic status [34]; this switch does not occur when delivery is by cesarean section.

We found here that in umbilical arterial serum of neonates born by spontaneous delivery, the concentration of cortisol was almost double that present in cord serum of neonates delivered by caesarean section. Accordingly, it was also found that the concentration of NEFA, glycerol and the ratio NEFA/glycerol were higher in umbilical arterial serum of neonates born by spontaneous delivery, indicating increased lipolysis of TAG, without differences in other lipid fractions such as total serum cholesterol, HDL-cholesterol or LDL-cholesterol. Following the same trend, the concentrations of adiponectin and RBP4, two of the most abundant adipocytokines produced by adipose tissue, were higher in cord serum of neonates born by spontaneous delivery. However, we did not find any difference in the concentrations of leptin in cord serum probably because the neonatal fat mass was similar in both groups, confirming the relationship between these two parameters [35]. The higher lipolytic activity in adipose tissue of neonates born by spontaneous delivery contribute to the higher cord blood serum concentrations of the most abundant individual fatty acids in their serum as well as the total saturated-, monounsaturated- and polyunsaturated- fatty acids. However, despite the significant higher lipolysis observed, we failed to find a positive relationship between any of these variables and the concentration of cord blood serum ANGPTL4, the concentrations of which were similar in both groups. This indicating that ANGPTL4 was not one of the signals involved in the activation of lipolysis at birth under the conditions of our study.

A major limitation of this study is the inability to measure directly the lipolytic activity of adipose tissue and therefore, to ensure that what we are considering as products of adipose tissue lipolysis, not actually come from the placenta. However, using the serum from the umbilical artery we minimize this possibility, since this mainly transport fetal metabolism products to the placenta [36]. Although fetal fatty acids, specially long-chain fatty acids, could proceed from placental transfer, it has been reported that this is not pertinent to the physiology during labor, and instead, the stress of labor leads to a mobilization of fetal lipid depots, probably as a consequence of raised glucocorticoid activity [34]. Moreover, although not studied in human, results from rats indicate that glycerol transport through the placenta is very low [37], so most of the arterial serum glycerol comes from lipolysis of fetal adipose tissue. Thus, the fact that umbilical arterial glycerol show a strong and significant correlation with most of serum concentrations of fatty acids in the group of infants born by spontaneous delivery but not those born by pre-labor cesarean, would corroborate the fetal origin of these lipolytic products.

The absence of differences in the concentrations of ANGPTL4 between the groups was unexpected for several reasons. On one hand, ANGPTL4 is a downstream target of the PPARα, PPARγ and PPARδ/β, so that its expression is induced by PPAR agonists in several types of cells and animal models [38]. Agonists for PPARα, γ and δ/β, including NEFAs, have been shown to increase the transcription of the ANGPTL4 gene and the concentration of ANGPTL4 in human plasma [17], [39]-[41]. Recently there have been indications that the expression of ANGPTL4 is also under hormonal control. Treatment with the synthetic glucocorticoid dexamethasone increased ANGPTL4 expression in vitro in HepG2 cells [19] and in primary cultures of hepatocytes and adipocytes, as well as in the livers and white adipose tissue of mice in vivo [8]. Moreover, it has been observed that under hyperinsulinemic conditions, such as hyperinsulinemic clamp in humans [19] and insulin treated mice [42], the expression and concentration of ANGPTL4 is reduced. These observations are interesting because increased fetal adrenal cortisol and decreased fetal insulin concentrations are two of the main signals that promote the mobilization of fatty acids from fetal fat depots and their subsequent oxidation in neonatal tissues with the onset of labor [21], [23]. However, in our study, despite the circulating NEFAs and cortisol in neonates born by spontaneous delivery being almost double and insulin being about half the concentrations observed in those delivered by cesarean section, serum neonatal ANGPTL4 did not show the increase in its concentration that would have been predicted from the previously published studies in adults.

Although our results appear to contradict the current wisdom on the relationship between ANGPTL4 and NEFA and glucocorticoids, it should be noted that plasma ANGPTL4 concentrations in humans are only increased by conditions associated with extreme elevated concentrations of NEFA, including intravenous infusion of lipid emulsion [11], [43], prolonged fasting [44] or prolonged caloric restriction [11], [44]; at baseline, none of these studies had found a significant correlation between NEFA and ANGPTL4 concentrations. In our population, the concentration of NEFA in cord serum at birth is around 100 µM, far below the concentrations observed in the other studies. We therefore propose that high concentrations of NEFA or glucocorticoids or both are required to induce the expression of ANGPTL4, and that these concentrations are not achieved at birth. It is also possible that the effect of NEFA and glucocorticoids on the expression of ANGPTL4 could be attenuated by other signals that participate in the increased lipolytic activity at birth, such as catecholamines and thyroid stimulating hormone [45], [46]. In these circumstances an additional stimulus may be required to induce the expression of ANGPTL4. This hypothesis is supported by our previous observation that neonates born by spontaneous delivery from pregnant women with gestational diabetes mellitus, with significantly higher concentrations in cord blood of cortisol (unpublished results), NEFAs and estimated insulin resistance (HOMA-IR) [47] than those found in neonates from control pregnant women, also showed a significantly higher concentration of ANGPTL4 in cord serum.

## Conclusions

In conclusion, in spite of the remarkable lipolytic activity in adipose tissue in neonates born by spontaneous delivery, the concentration of ANGPTL4 remained unchanged compared to that found in neonates born by pre-labor cesarean section. The higher concentrations of cortisol and especially of NEFA observed in arterial cord serum of neonates born by spontaneous delivery did not induce the increase in the levels of ANGPTL4 reported to be present in those conditions of adults that are accompanied by an acute raise of NEFA. These results support the hypothesis that the regulation of circulating ANGPTL4 concentrations in vivo is more complex than is currently understood, with potential involvement of additional regulatory inputs.
